# The HPV16 E6 binding protein Tip-1 interacts with ARHGEF16, which activates Cdc42

**DOI:** 10.1038/sj.bjc.6606026

**Published:** 2010-12-07

**Authors:** A W Oliver, X He, K Borthwick, A J Donne, L Hampson, I N Hampson

**Affiliations:** 1 University of Manchester Gynaecological Oncology Laboratories, School of Cancer and Enabling Sciences, St Mary's Hospital, Oxford Road, Manchester M13 9WL, UK

**Keywords:** HPV16 E6, Tip-1, ARHGEF16, Rho proteins and Cdc42

## Abstract

**Background:**

Guanidine exchange factor (GEF)-catalysed activation of Rho proteins such as Cdc42 has been shown to have a crucial role in cellular transformation, malignant progression and invasion. We have previously shown that the HPV16 E6 oncoprotein binds to the PDZ domain protein Tax-interacting-protein 1 (Tip-1) and we now report identification and functional analysis of a novel Tip-1 binding GEF.

**Methods:**

Yeast two-hybrid, *in vitro* pull-down, site-directed mutagenesis, semiquantitative PCR, co-immunoprecipitation and western blotting were used to identify/confirm novel Tip-1 binding partners and analyse cellular expression levels. *In vitro* kinetic analyses of recombinant proteins, siRNA gene silencing and in cell assays were used to measure Rho protein activation.

**Results:**

Tax-interacting-protein 1 was shown to interact with ARHGEF16 by its carboxyl PDZ binding motif. Levels of ARHGEF16 were increased in transformed and immortalised cells expressing ectopic HPV16 E6 and Cdc42 was co-immunoprecipitated by ARHGEF16 in the presence of high-risk HPV E6. *In vitro* kinetic analysis confirmed that recombinant ARHGEF16 activates Cdc42 and this was increased by the addition of recombinant Tip-1 and E6. Cells expressing HPV16 E6 had higher levels of Cdc42 activation, which was decreased by siRNA silencing of either Tip-1 or ARHGEF16.

**Conclusion:**

These data suggest that HPV16 E6, Tip-1 and ARHGEF16 may cooperate to activate Cdc42 and support a potential link between the expression of HPV16 E6 and Cdc42 activation.

The E6 oncoprotein of high-risk HPV type 16 has been shown to be crucial for the transforming properties of the virus. To further understand how it may elicit these tumorigenic effects, we have studied the cellular biological pathways that it targets. As a result of this, we have previously reported a novel interaction of HPV16 E6 with the class 1 PDZ domain protein Tip-1 (Hampson *et al*, 2004). Several PDZ domain-containing proteins have been documented to interact with E6 via its carboxyl terminal PDZ binding motif. Indeed, this motif has been shown to be essential for many of its oncogenic properties (Kiyono *et al*, 1997; Nguyen *et al*, 2003; Spanos *et al*, 2008; Wise-Draper and Wells, 2008). Interestingly, the downstream consequence of E6 binding is usually proteasomal degradation of the target PDZ protein, but we have clearly shown that this is not the case for Tip-1 (Hampson *et al*, 2004). Tax-interacting-protein 1 was found to be necessary for E6-dependent increased cell motility, a hallmark of transformation, which could be inhibited by the Rho kinase inhibitor Y27632 (Hampson *et al*, 2004). As Tip-1 has also been shown to bind to the carboxyl terminal PDZ binding motif of the human T-cell leukaemia virus (HTLV1) Tax oncoprotein (Rousset *et al*, 1998), there is good evidence that Tip-1 may have a role in viral carcinogenesis.

In support of this, other studies have shown that Tip-1 is involved in both Rho A and Wnt signalling (Reynaud *et al*, 2000; Kanamori *et al*, 2003), which are two crucial pathways involved in the neoplastic process (Hampson *et al*, 2004; Hall and Fujii, 2005; Lichtig *et al*, 2010). As Tip-1 has been shown to exist as a dimer in solution (Aledo *et al*, 2001), this raises the possibility that Tip-1 may form a link between E6 and components of these pathways. This would be consistent with the proposed ability of viral oncoproteins to promote transcriptional re-programming by modifying Rho and Wnt protein signalling. Recent work has also shown that Tip-1 binds to the apoptosis mediator FAS and undergoes specific conformational rearrangements upon binding to this and its other ligands (Banerjee *et al*, 2008). These authors state that Tip-1 regulates signalling pathways through its PDZ domain and deregulation of any of these can lead to the development of cancer. It is also worth noting that they conclude that identification and characterisation of the protein–protein interactions of Tip-1 will be critical to understanding how it regulates cellular dynamics.

As a continuation of our work on E6 and Tip-1 (Hampson *et al*, 2004), we now describe the results of screening for novel Tip-1 binding partners. This has identified the guanine nucleotide exchange factor 16 (ARHGEF16; subsequently abbreviated to GEF16). Guanidine exchange factors are known to regulate the activation of Rho-like GTPases (Rossman *et al*, 2005), and when this occurs inappropriately, they can aid malignant transformation (Fritz and Kaina, 2006). We have previously shown that ectopic expression of GEF16 can transform NIH3T3 cells (Hampson *et al*, 2009) and we now show the interaction of Tip-1 with GEF16 combined with preliminary functional characterisation of this protein.

## Materials and methods

### Yeast two-hybrid

Tax-interacting-protein 1 was cloned into the yeast vector pEG202 (Origene Technologies, Rockville, MD, USA) and used as bait to carry out yeast two-hybrid screening and mating assays as described previously (Hampson *et al*, 2004).

### Expression of GST-Tip fusion protein

The Tip-1 open-reading frame (ORF) was cloned into the pGEX-2T vector (GE Healthcare, Little Chalfont, Bucks, UK), expressed as a glutathione *S* transferase (GST) fusion protein in *Escherichia coli* XL1 cells (Stratagene, Amsterdam, the Netherlands) and captured on glutathione-sepharose (GE Healthcare) according to the manufacturer's instructions.

### Cloning of GEF16 ORF and construction of GEF16 PDZ deletion mutant

The 392–1657 bp GEF16 ORF from NM_014448 was PCR amplified with hi-fidelity *Taq* polymerase (Roche Diagnostics Ltd, Mannheim, Germany) from a placental cDNA library using the following primers: GEF16 forward, 5′-GCCATGTTCGAGATCCTCACGT-3′ GEF16 reverse, 5′-AACCATGCTGGGTCCTTGAGAC-3′.

The PCR product was cloned into the pCR4-TOPO vector (Invitrogen, Paisley, UK) and the sequence fidelity verified. This was then used as a PCR template for generating wild-type GEF16: *wt*GEF16 forward, 5′-CTCGGATCCATGTTCGAGATCCTC-3′ *wt*GEF16 reverse, 5′-AACCATGCTGGGTCCTTGAGAC-3′ and PDZ deletion mutant GEF16: *mut*GEF16 forward, 5′-CTCGGATCCATGTTCGAGATCCTC-3′ *mut*GEF16 reverse, 5′-CTCGAATTCCTACTCCACCCGCAGACG-3′.

These products were then cloned into the expression vector pCITE-4A (Merck Chemicals Ltd, Beeston, Notts, UK) using the incorporated *Bam*HI and *Eco*RI restriction sites.

### *In vitro* transcription translation of GEF16 and GEF16 PDZ deletion mutant

The GEF16 pCITE-4A constructs were used with an *in vitro* transcription translation (IVTT) reticulocyte lysate system (Promega, Southampton, UK) according to the manufacturer's instructions to produce S-tagged recombinant GEF16 proteins.

### GST pull-down assay

Extract pre-clearing was carried out by incubating 1 ml of binding buffer (1 × PBS, 0.1% NP40, 0.5 mM DTT, 10% glycerol, 1 mM PMSF and 2 *μ*g/ml aprotinin) 40 *μ*l of glutathione-sepharose beads (GE Healthcare) and 80 *μ*l of IVTT product at 4°C. Following centrifugation, the pellet was discarded and the IVTT supernatant divided into two aliquots. Four micrograms of GST control or GST-Tip-1 protein-bound beads were added to separate aliquots of pre-cleared supernate and these were incubated at 4°C for 2 h, centrifuged as before and the pellet washed 5 × with binding buffer. The beads were then re-suspended in Laemmli buffer (20% glycerol, 2% SDS, 25 *μ*g/ml bromophenol blue, 125 mM Tris-HCl (pH 6.8)) heated to 85°C, separated by SDS–PAGE, western blotted and immunoprobed with anti-GEF16.

### Cell culture, stable gene transfection and siRNA silencing

Human HPV-negative C33A cervical cancer cells were cultured and stably transfected with complete ORFs of E6 from low-risk HPV6 (C33AT6 E6) and high-risk HPV16 (C33AT16 E6) as described previously (Hampson *et al*, 2004; Donne *et al*, 2007). Non-transformed human telomerase immortalised keratinocytes (hTert) cells were routinely cultured as described in Dickson *et al* (2000) and were stably transfected with either high-risk (hTertT16 E6) or low-risk E6 (hTertT6 E6) as described previously (Donne *et al*, 2009). Both polyclonal and monoclonal cell lines were derived and expanded in the presence of G418.

Four different siRNAs each for both GEF16 and Tip-1 plus an ‘AllStars’-negative control with no significant off-target homology were designed by and obtained from Qiagen (West Sussex, UK; Flexitube). These were transiently transfected into hTertT16 E6 cells using Lipofectamine 2000 according to the manufacturer's instructions (Invitrogen), and GEF16 and Tip-1 mRNA downregulation was assessed by RT–PCR 48 h post-transfection. Using the identical procedure and culture conditions, hTertT16 E6, hTertT6 E6 and vector control hTert cells were then transfected in triplicate with the GEF16 and Tip-1 siRNA oligonucleotides, which produced the greatest degree of gene silencing and the AllStars-negative control siRNA. After 48 h, protein extracts were prepared from these cultures for the G-LISACdc42 Activation Assay Biochem Kit colorimetric Exchange Assay (see ‘In cell-activated Cdc42 assays’) as per the manufacturer's instructions. Every data point was the result off three separate assays from three separately transfected culture dishes.

### RT–PCR

Total cellular RNAs were prepared using the SuperScript III Cells Direct cDNA Synthesis Kit as recommended by the manufacturer (Ambion, Cambridgeshire, UK). Total RNAs from tissue samples were isolated using Trizol Reagent (Invitrogen). All DNaseI-treated RNAs were then reverse transcribed with random decamers. Polymerase chain reaction was performed in 20 *μ*l of a reaction mixture containing 2 *μ*l of reverse-transcribed product, 10 *μ*l of 2 × Bio-Red and 0.1 *μ*M of each primer. The specific primer pairs used were as follows: GAPDH forward, 5′-CATTGACCTCAACTACATGGT-3′, GAPDH reverse, 5′-TCGCTCCTGGAAGATGGTGAT-3′ *β*-actin forward, 5′-ATGGGTCAGAAGGATTCCTA-3′, *β*-actin reverse, 5′-ATCACGATGCCAGTGGTAC G-3′ Tip-1 forward, 5′-CCGTGGTGCAAAGAGTTGAAA-3′, Tip-1 reverse, 5′-GTGTGTGACCATGGTCATGTC-3′ GEF16 forward, 5′-GAGTTCTCCTACCAGCACAG-3′, GEF16 reverse, 5′-AGGATCAGGAAGGAGAGCAT-3′ and HPV16 E6 forward, 5′-AATGTTTCAGGACCCACAGG-3′, HPV16 E6 reverse, 5′-CATACAGCATATGGATTCCC-3′.

The reaction mixture was denatured at 94°C for 4 min and amplified between 30 and 33 cycles of 30 s denaturation at 94°C, 30 s annealing at 53–55°C and 30 s extension at 72°C, followed by a single 5-min extension at 72°C.

### Competitive RT–PCR

Two microlitres of the RT product was co-amplified with a constant amount of the competitive template in a final volume of 20 *μ*l PCR mixture containing 10 *μ*l of 2 × Bio-Red (Bioline, London, UK) and 0.1 *μ*M of each primer. The reaction mixture was amplified as described previously. The PCR products were separated by 1.5% agarose gel electrophoresis and stained with ethidium bromide. The intensity of DNA bands was quantified using the ImageJ 1.38 software.

### Antibodies used

The primary antibodies used were as follows: Tip-1, 1 : 100 (Abnova, Heidelberg, Germany); GEF16, 1 : 100 (Abnova); RhoA, 1 : 500 (Pierce Biotechnology, Rockford, IL, USA); Rac1, 1 : 2000 (Abcam, Cambridge, UK); GAPDH, 1 : 1500 (Abcam); Cdc42, 1 : 200 (Cell Biolabs Inc., San Diego, CA, USA); and *β*-actin 1 : 1500 westerns and 1 : 1000 immunostaining (Sigma, Dorset, UK). Secondary antibodies (DAKO Cytomation, Denmark) were HRP conjugated for western applications and used at 1 : 2000.

### Immunoprecipitation and western blotting

For immunoprecipitation, cell lysates were prepared with Cellytic-M Cell Lysis (Sigma-Aldrich, Poole, UK) as recommended by the manufacturer. Immunocomplexes were precipitated from 1.5 ml of prepared cell lysate supernatants (equal concentrations) using 2 *μ*g of anti-GEF16 and 50 *μ*l of protein G-sepharose (Sigma-Aldrich) for 48 h at 4°C. After two sequential washes using PBS–0.1% Tween 20, the resulting pellets were heated for 3 min at 85°C in 2 × Laemmli buffer (with DTT) and separated by SDS–PAGE. Western immunoblotting was carried out a described previously (Hampson *et al*, 2004).

### Rho/GEF exchange assays

Recombinant GEF16 (GenWay Biotech, San Diego, CA, USA), Tip-1 (made in-house, see previous) and HPV16 E6 (Insight Biotechnology Limited, Wembley, UK) were used in the RhoGEF exchange assay kit (Cytoskeleton, Denver, CO, USA). Two-micromolar concentrations of GEF16, Tip-1 and HPV16 E6 were assayed against RhoA, Rac1 and Cdc42 according to the manufacturer's instructions using a Spectra Max Gemini XS Microplate Spectrofluorometer (Molecular Devices, Sunnyvale, CA, USA).

### In cell-activated Cdc42 assays

Levels of activated Cdc42 were initially assessed in non-transformed hTertT16 E6 and hTertT6 E6 keratinocytes using a Cdc42 PAK1 binding activation assay kit (Cell Biolabs Inc.) according to the manufacturer's instructions. This was superseded by the more accurate colorimetric G-LISACdc42 Activation Assay Biochem Kit (Cytoskeleton), which was used according to the manufacturer's instructions. Each data point represents three separate assays.

## Results

### Identification and confirmation of GEF16 as a Tip-1 binding protein

**Figure 1:**
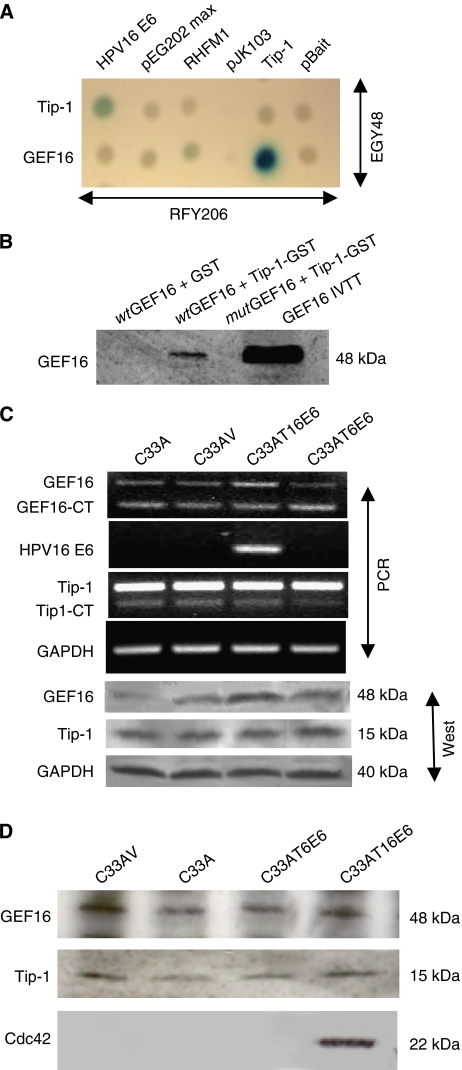
Interactions, regulation and stability of GEF16. (**A**) Yeast mating assay confirming that Tip-1 binds to short carboxyl terminal GEF16 fragments isolated from the primary Tip-1 yeast two-hybrid screen. (**B**) Glutathione transferase pull-down of Tip-1 with IVTT full-length wild-type and mutant GEF16 (*wt*GEF16/*mut*GEF16). The *wt*GEF16 contains the PDZ-binding domain ETDV, whereas *mut*GEF16 has the TDV residues deleted. Tip-1-GST fusion protein and GST control protein were bound to glutathione-sepharose beads. These were mixed with equal amounts of the IVTT *wt*GEF16 or *mut*GEF16 proteins, and then washed with binding buffer. Bound proteins were separated by SDS–PAGE and visualised by western immunoblotting with anti-GEF16. The GST-Tip-1 protein bound to the *wt*GEF16, but did not bind to the mutant form, whereas the GST control did not bind either product. (**C**) Competitive template RT–PCR and western blot analysis of GEF16 and Tip-1 expression in mRNAs and proteins extracted from C33A, C33AV, C33AT16 E6 and C33AT6 E6 cells. mRNA's were reverse transcribed and the resulting cDNAs PCR amplified using primers specific for GAPDH, HPV16 E6, GEF16 and Tip-1 by competitive template PCR. Total proteins were extracted from the same cells, separated by SDS–PAGE, electroblotted and immunoprobed with anti-GEF16 and anti-Tip-1. Anti-GAPDH was used as a loading control. (**D**) Immunoprecipitation with anti-GEF16 from lysates of C33AV, C33AT16 E6 and C33AT6 E6 cells treated with 10 *μ*M of the selective proteasome inhibitor MG132 for 4 h. When immunoprobed with Tip-1 and Cdc42, it can be seen that Tip-1 is associated with GEF16 in both the presence and absence of T16 E6. Cdc42 was detected in association with the GEF16 complex in the presence of HPV type 16 E6, but not in type 6 or vector and parent control cells. (GEF16 has a putative Cdc42 binding site at amino acids 385–391 (QRTLQKL)).

To elucidate potential cellular binding partners for Tip-1, a LexA yeast two-hybrid screen was carried out using Tip-1 as bait. This identified four clones that were homologous to GEF16, a member of the Dbl family of Rho GEFs (accession no. BC002681). All the yeast clones were incomplete and corresponded to nucleotides 1216–1657 at the 3′ end of the GEF16 coding sequence. The interaction between the GEF16 fragment and Tip-1 was further confirmed in yeast by a mating assay (Figure 1A). This was not possible with the full-length 422 amino acid GEF16 protein, as expression in yeast proved refractory owing to protein instability.

To confirm the GEF16/Tip-1 interaction outside the yeast system, both full-length *wt*GEF16 and a deletion *mut*GEF16 that lacked the carboxyl terminal PDZ binding motif were produced by IVTT. The Tip-1 protein was expressed in the bacteria as a GST fusion and this was used in a GST pull-down assay with the *wt*GEF16 and *mut*GEF16 products (Figure 1B). The GST-Tip-1 protein associated with the *wt*GEF16 but did not bind to the *mut*GEF16 form. The GST control, as expected, did not bind to either. This indicates that GEF16 most likely interacts with Tip-1 via its carboxyl-terminal PDZ domain.

### Expression of HPV16 E6 in transformed cells induces a moderate upregulation of GEF16 mRNA and protein

As Tip-1 had been previously shown to interact with HPV16 E6 (Hampson *et al*, 2004), the effects of constitutive ectopic expression of high-risk E6 proteins on the level of GEF16 mRNA in C33A cells was investigated by semiquantitative competitive template PCR (Fandrey and Bunn, 1993). A low-risk type 16 E6-expressing cell line was used as an additional control, as this lacks the PDZ binding motif found in high-risk E6s. The absence of this feature in low-risk E6 is thought to contribute to its reduced tumorigenicity. The results showed that in the presence of high-risk E6, there was a modest increase in GEF16 mRNA expression when compared with the parent, vector and low-risk E6-transfected cells (Figure 1C). Anti-GEF16 and Tip-1 antibodies were then used to immunoprobe a western blot of proteins extracted from the same cells. This supported the RNA data and confirmed an increase in the level of GEF16 protein in C33AT16 E6 cells, whereas Tip-1 expression remained constant across all cells tested (Figure 1C).

### GEF16 co-immunoprecipitates with Tip-1 from MG132-treated C33AV and C33AT16 E6 cells and differentially associates with Cdc42

Immunoprecipitation of detectable amounts of GEF16 initially proved difficult, which suggested that the protein could have a high rate of turnover. Previous studies have indicated that GEFs can be unstable (Hayakawa *et al*, 2005) and proteasomal degradation has been identified as a regulatory mechanism for some Rho GEFs such as the proto-oncogene Dbl (Kamynina *et al*, 2007). Also like Dbl, GEF16 has a stretch of proline, glutamic acid, serine and threonine residues (PEST sequence) at residues 222–246. These sequences have been implicated in the rapid turnover of proteins (Rogers *et al*, 1986). Proline, glutamic acid, serine and threonine sequences with PEST-FIND scores higher than +5 are thought to be the best candidates for degradation signals (Rechsteiner and Rogers, 1996), and as the GEF16 PEST has a score of +13.77, it is very likely to possess this property (see http://emboss.bioinformatics.nl/cgi-bin/emboss/epestfind). In light of these observations, it was decided to assess the effects of pretreating the C33A cultures with the proteasome inhibitor MG132 to increase the stability of GEF16. Following MG132 treatment, immunoprecipitated GEF16 was found to co-precipitate with Tip-1 in C33AV and C33AT16 E6 cells, but not low-risk C33AT6 E6. Further analysis of GEF16 immunoprecipitates showed no association with the Rho proteins RhoA and Rac (data not shown), but Cdc42 was detected in immunoprecipitates from C33AT16 E6 cells. This provides evidence that, in the presence of high-risk HPV16 E6, GEF16 can associate with Cdc42 (Figure 1D).

### GEF16 activates Cdc42 *in vitro*

**Figure 2:**
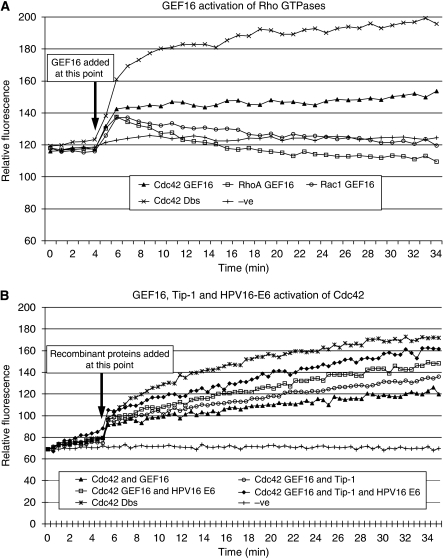
Guanidine exchange factor 16 activates Cdc42 *in vitro*. (**A**) Fluorescent kinetic analysis of the ability of recombinant GEF16 to activate Cdc42, RhoA and Rac1 *in vitro.* Two micromolar concentrations of GEF16 recombinant protein were added in triplicate to each Rho GTPase being assayed at 4 min after initial readings had been taken with the Rho proteins and assay buffers alone. After the addition of GEF16, it can clearly be seen that there is significant activation of Cdc42 and to a lesser extent Rac1. (**B**) Fluorescent kinetic analysis of Cdc42 activation by GEF16 in the presence of high-risk HPV16 E6 and Tip-1. Different combinations of 2 *μ*M concentrations of the recombinant proteins GEF16, Tip-1 and HPV16 E6 were added after 4 min of initial readings being taken with Cdc42 and assay buffers alone. Each combination was carried out in triplicate. It can be seen that Cdc42 activation by GEF16 is enhanced in the presence of Tip-1 and E6.

The finding that in the presence of high-risk type 16 E6 Cdc42 could co-precipitate with GEF16 (see Figure 1D), coupled with the presence of a potential Cdc42 binding motif in the GEF16 primary sequence (see Discussion), suggested that GEF16 could potentially function as an activator of Cdc42. To address this issue, an *in vitro* fluorescent kinetic analysis of Rho protein activation was carried out. The addition of recombinant GEF16 protein to Cdc42 produced a marked increase in the transfer and exchange of GTP for GDP bound to Cdc42 at approximately half the rate seen with the Dbs GEF-positive control (Figure 2A). GEF16 also caused a modest activation of Rac1 and no activation of RhoA (Figure 2A). (It should be noted that the Dbs-positive control used in the kit is known by the manufacturers to have reduced activity with Rac1.)

### GEF16, Tip-1 and HPV16 E6 cooperate to activate Cdc42 *in vitro*

The same *in vitro* fluorescent kinetic assay was used to analyse GEF16-catalysed Cdc42 activation in the presence of recombinant Tip-1 and/or HPV16 E6. The addition of the Tip-1 protein plus GEF16 produced a modest increase in the GTP activation of Cdc42 above that produced by GEF16 alone. The addition of HPV16 E6 plus GEF16 induced a more pronounced activation of Cdc42, whereas addition of all three recombinant proteins produced the greatest increase in Cdc42 activation observed (Figure 2B).

### Expression of HPV16 E6 is associated with the upregulation of GEF16 in non-transformed human keratinocytes (hTert cells)

**Figure 3:**
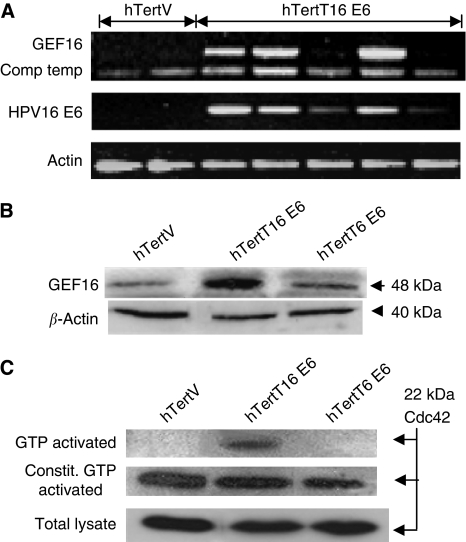
Expression of HPV16 E6 is associated with the upregulation of GEF16 and higher levels of activated Cdc42 in non-transformed cells. (**A**) Competitive template RT–PCR analysis of GEF16 levels in mRNAs, extracted from hTertV and hTertT16 E6 cells. mRNA was extracted, reverse transcribed and the resulting cDNAs amplified with primers specific for *β*-actin, HPV16 E6 and competitive template primers specific for GEF16. (**B**) Western immunoblot of proteins extracted from hTert cells stably transfected with either HPV16 E6, HPV6 E6 or vector control. *β*-Actin was used as a loading control. (**C**) Western immunoblot analysis of GTP-activated Cdc42 in HPV16 E6- and HPV6 E6-transfected hTert cells. hTertT16 E6 cells showed increased levels of activated Cdc42. The addition of GTP to the PAK1 Cdc42 assay shows constitutive GTP activation of the available Cdc42.

As C33A cells are a transformed cell line, it was decided to analyse GEF16 expression in non-transformed E6-transfected human keratinocyte cultures (hTert cells). Competitive template RT–PCR analysis showed that GEF16 mRNA was upregulated in several different monoclonal high-risk E6-expressing hTert cell lines (hTertT16 E6) when compared with vector control cells (Figure 3A). Significantly, the level of E6 mRNA detected in each clone appears to correlate with the level of GEF16 mRNA. Western blot analysis of protein extracts from a pooled polyclonal population of these hTert derivative cell lines showed that upregulated expression of the GEF16 protein was specifically found associated with cells expressing the T16 E6 protein (Figure 3B).

### hTertT16 E6 cells have higher levels of activated Cdc42 than hTertT6 E6 and vector control hTert cells

As the previous *in vitro* data suggested that GEF16 could activate Cdc42, the levels of activated Cdc42 were compared between E6-expressing and control hTert cells. Western blot analysis of PAK1-captured, GTP-bound, Cdc42 from cell lysates clearly showed higher levels of activated Cdc42 are present in hTertT16 E6 cells (Figure 3C) when compared with hTertT6 E6 and vector control cells. These lysates were also screened for Rac1, but no detectable signal was seen; thus, the data have not been included

### siRNA silencing of GEF16 and Tip-1 markedly reduces CDc42 activation in hTertT16 E6 cells

**Figure 4:**
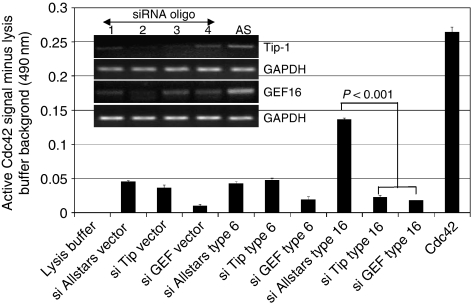
HPV16 E6-dependent upregulation of Cdc42 activation depends on the expression of Tip-1 and GEF16. Human telomerase immortalised keratinocyte T16 E6 cells were transiently transfected with four different Tip-1 and GEF16 siRNAs (oligos 1–4) plus the AllStars (AS)-negative control to assess the optimal siRNAs. RT–PCR analysis of mRNA levels 48 h post-transfection showed that oligo 2 was the most effective in each case. (Tip-1 oligo 2 sequence: 5′-CAAGACGGACAAGGGTATTTA-3′ GEF16 oligo 2 sequence: 5′-CACGTCGGAGTTCTCCTACCA-3′.) For the main experiment, cells were transiently transfected with Tip-1 oligo 2, GEF16 oligo 2 and the AllStars-negative control, and the colorimetric Cdc42 activation assay carried out 48 h later. All cell extracts were adjusted to 0.25 mg ml^−1^ of protein for the assay. AllStars-negative control siRNA-transfected hTertT16 E6 cells had the highest endogenous level of activated Cdc42, which was markedly reduced by silencing of Tip-1 or GEF16 expression (*P*<0.001).

To confirm the observed T16 E6-dependent increase in Cdc42 activation shown in Figure 3C, an alternative and more sensitive, colorimetric ELISA-based Cdc42 activation assay was used to assess Cdc42 activation. This was also used to evaluate the effects of siRNA silencing of Tip-1 or GEF16 on Cdc42 activation. Tax-interacting-protein 1 siRNA oligo 2 and GEF16 siRNA oligo 2 were found to be the most effective at silencing their respective mRNAs and were subsequently used to evaluate the effects of this procedure on Cdc42 activation (Figure 4). The results verified that Cdc42 activation was consistently higher in the AllStars siRNA control oligo-transfected hTertT16 E6 cells than in either AllStars-transfected hTertT6 E6 or vector cells. Silencing of Tip-1 produced no significant difference in the extent of Cdc42 activation found in low-risk E6 hTertT6 E6 or hTert vector cells, whereas silencing of GEF16 reduced Cdc42 activation in both these cell types. However, silencing of either Tip-1 or GEF16 in high-risk E6 hTertT16 E6 cells produced a pronounced reduction of Cdc42 activation when compared with the AllStars control (Figure 4). None of the siRNAs used produced any discernible cell death or had any effect on cell growth characteristics.

## Discussion

In this study, we have shown that the HPV16 E6 binding protein Tip-1 (Hampson *et al*, 2004) interacts with the Dbl family member GEF16 via its carboxyl PDZ binding sequence and that ectopic expression of high-risk, but not low-risk, E6 is associated with a modest upregulation of GEF16 in both transformed and non-transformed cells. It is significant that overexpression of GEFs can promote malignant transformation and progression (Rossman *et al*, 2005; Fritz and Kaina, 2006), and we have previously shown that ectopic expression of GEF16 transforms NIH3T3 cells (Hampson *et al*, 2009).

Guanidine exchange factors promote the GTP activation of Rho GTPase's by catalysing the exchange of GDP for GTP. The 48-kDa GEF16 protein reported here is one of the smallest Dbl-related GEFs containing one *src* homology 3 domain in addition to the minimal structural unit (PH/DH domains) and a PDZ binding motif at the C-terminal (accession no. NM_014448.3). Bioinformatic analysis indicated that GEF16 has a putative Cdc42 binding sequence QRTLQKL located in its DH domain (Blanke and Jackle, 2006), which prompted us to investigate its ability to activate this particular Rho protein.

Initial evidence supporting this hypothesis was provided by the observation that GEF16 co-immunoprecipitated with both Tip-1 and Cdc42, although this was only found when high-risk E6 was also present and when cells were pretreated with the proteasome inhibitor MG132. In the absence of MG132, GEF16 did not co-precipitate with Tip-1 in either control or E6-expressing cells, which suggests that proteasomal degradation may influence this interaction. Indeed, MG132 is known to stabilise the interaction of the Rho-GEF Pbl/ECT2 with the E3 ligase E6AP (Reiter *et al*, 2006), and the suggestion that GEF levels may be regulated by the proteasome has been previously reported by others. Examples include the Cdc42-specific GEF h-PEM2, which is subject to proteasomal degradation via the HECT-type E3 ligase Smurf1 (Yamaguchi *et al*, 2008) and the Cdc42-specific GEF FWD1, which is stabilized in the presence of MG132 (Hayakawa *et al*, 2005). Indeed, we have shown that the full-length GEF16 protein is unstable in yeast cells and it contains PEST sequences, which have previously been shown to signal rapid degradation in yeast (Marchal *et al*, 1998).

In light of these stability problems, we initially opted to analyse the ability of recombinant GEF16 to activate Rho proteins using a kinetic *in vitro* system. This showed that GEF16 did not activate Rho A, had a modest ability to activate Rac-1, but showed the largest activity with Cdc42. These experiments were then extended to evaluate the effects of adding recombinant GEF16 plus Tip-1, and GEF16 plus Tip-1 and HPV16 E6 to the Cdc42 assay system, with the result that the greatest activation was observed when all three proteins were present. This *in vitro* assay system has been used very successfully by others (Guilluy *et al*, 2010), but, as it is based on the use of recombinant proteins, we employed two additional in-cell test systems to establish whether these effects occurred with native proteins *in situ*. Both of these in-cell assays showed that high-risk E6-expressing cells had the greater levels of Cdc42 activation when compared with vector control or low-risk T6 E6-expressing cells. Furthermore, since silencing expression of either Tip-1 or GEF16 produced a highly significant (*P*=0.001) reduction in Cdc42 activation in T16 E6-expressing cells, this provides clear evidence that these two proteins are involved with the ability of T16 E6 to upregulate Cdc42 activation in cells. The observation that silencing Tip-1 has little effect on Cdc42 activation in T6 E6 and vector control cells reinforces the conclusion that T16 E6 upregulates Cdc42 activation through both Tip-1 and GEF16.

While the interplay between GEF16, Tip-1 and E6 and the subsequent alterations in activated Cdc42 levels are clearly complex and still require further elucidation, the results presented suggest that GEF16-dependent activation of Cdc42 may be upregulated by high-risk E6. One potential explanation for these findings may be the uniqueness of Tip-1 as a PDZ protein in that it contains only a single class 1 PDZ domain. It is known that the majority of PDZ proteins contain multiple protein–protein interaction domains, which act as molecular scaffolds important for a variety of cellular functions including cell signalling. The Tip-1 protein is known to form dimers in solution (Aledo *et al*, 2001) and it has been reported to be a negative regulator of PDZ-based scaffolding assemblies (Alewine *et al*, 2006). Therefore, it is possible that the binding of HPV16 E6 to the Tip-1 PDZ domain may affect its ability to interact with and regulate a signalling pathway involving GEF16 and Cdc42, resulting in an increase in activated Cdc42 levels. Indeed, this is supported by the previously reported interactions of Scrib, *β*PIX and Cdc42. Scrib is a multidomain LRR/PDZ scaffold protein, which controls the localisation and activation of Cdc42 via its interaction with *β*PIX, which is a Cdc42-activating GEF (Osmani *et al*, 2006). Furthermore, it is highly significant that HPV16 E6 has also been shown to interact with Scrib, yet, unlike Tip-1, this results in its proteasomal degradation (Nakagawa and Huibregtse, 2000). Thus, it is hypothesised that the interaction of high-risk E6 with PDZ proteins, such as Scrib and Tip-1, may cooperate to perturb the delicate balance of interactions between GEFs (*β*PIX and/or GEF16) with Cdc42. This coordinated assault by E6 ultimately leads to deregulated control of Cdc42 activation.

Our data are not without precedent since it is well known that other viral oncoproteins can affect Rho protein function. For example, HTLV1 Tax, which interestingly like HPV high-risk E6 also interacts with Tip-1, has been shown to interact directly with Rac, Rho and most significantly Cdc42 (Wu *et al*, 2004). Similarly, the Epstein–Barr virus LMP1 oncoprotein can promote the activation of Cdc42 signalling (Eliopoulos and Young, 2001).

Therefore, in summary our data provide evidence that there are pathways connecting the expression of the high-risk HPV type 16 E6 protein to enhanced Cdc42 activation and Tip-1 and GEF16 function as components of this system. The exact nature of the interplay between these proteins and the precise molecular mechanisms by which they elicit Cdc42 activation are worthy of further investigation.
